# Experimental Assessment of Leptomeningeal Metastasis Diagnosis in Medulloblastoma Using Cerebrospinal Fluid Metabolomic Profiles

**DOI:** 10.3390/metabo11120851

**Published:** 2021-12-07

**Authors:** Ji Hye Im, Byong Chul Yoo, Jun Hwa Lee, Kyue-Yim Lee, Kyung-Hee Kim, Jong Heon Kim, Hyeon Jin Park, Meerim Park, Sang Hyeon Lee, Ji-Woong Kwon, Sang Hoon Shin, Heon Yoo, Jeyul Yang, Seung Ah Choi, Seung-Ki Kim, Ho-Shin Gwak

**Affiliations:** 1 Department of Cancer Control, Graduate School of Cancer Science and Policy, National Cancer Center, Goyang 10408, Korea; 75262@ncc.re.kr (J.H.I.); 70564@ncc.re.kr (K.-Y.L.); 2 Department of Cancer Biomedical Science, Graduate School of Cancer Science and Policy, National Cancer Center, Goyang 10408, Korea; yoo_akh@ncc.re.kr (B.C.Y.); jhkim@ncc.re.kr (J.H.K.); 3 Cancer Diagnostics Branch, Division of Clinical Research, Research Institute, National Cancer Center, Goyang 10408, Korea; jhlee@ncc.re.kr (J.H.L.); kyunghee@ncc.re.kr (K.-H.K.); 4 Proteomics Core Facility, Research Core Center, Research Institute, National Cancer Center, Goyang 10408, Korea; 5 Cancer Molecular Biology Branch, Division of Cancer Biology, Research Institute, National Cancer Center, Goyang 10408, Korea; 6 Department of Pediatrics, Center for Pediatric Cancer, National Cancer Center, Goyang 10408, Korea; hjpark@ncc.re.kr (H.J.P.); meerim@ncc.re.kr (M.P.); 7 Department of Radiology, National Cancer Center, Goyang 10408, Korea; shlee@ncc.re.kr; 8 Neuro-Oncology Clinic, National Cancer Center, Goyang 10408, Korea; jwkwon@ncc.re.kr (J.-W.K.); nsshin@ncc.re.kr (S.H.S.); heonyoo@ncc.re.kr (H.Y.); nsjeyul@ncc.re.kr (J.Y.); 9 Department of Neurosurgery, Seoul National University Hospital, Seoul National University College of Medicine, Seoul 03080, Korea; aiippo7@gmail.com

**Keywords:** cerebrospinal fluid, leptomeningeal metastasis, magnetic resonance image, medulloblastoma, metabolome profile

## Abstract

Diagnosing leptomeningeal metastasis (LM) in medulloblastoma is currently based on positive cerebrospinal fluid (CSF) cytology or magnetic resonance imaging (MRI) finding. However, the relevance of discordant results has not been established. We evaluated the diagnostic potential of CSF metabolomic profiles in the medulloblastoma LM assessment. A total of 83 CSF samples from medulloblastoma patients with documented MRI and CSF cytology results at the time of sampling for LM underwent low-mass ions (LMIs) analysis using liquid chromatography-mass spectrometry. Discriminating LMIs were selected by a summed sensitivity and specificity (>160%) and LMI discriminant equation (LOME) algorithms, evaluated by measuring diagnostic accuracy for verifying LM groups of different MRI/cytology results. Diagnostic accuracy of LM in medulloblastoma was 0.722 for cytology and 0.889 for MRI. Among 6572 LMIs identified in all sample, we identified 27 discriminative LMIs differentiating MRI (+)/cytology (+) from MRI (−)/cytology (−). Using LMI discriminant equation (LOME) analysis, we selected 9 LMIs with a sensitivity of 100% and a specificity of 93.6% for differentiating MRI (+)/cytology (+) from MRI (−)/cytology (−). Another LOME of 20 LMIs significantly differentiated sampling time relative to treatment (*p* = 0.007) and the presence or absence of LM-related symptoms (*p* = 0.03) in the MRI (+)/cytology (−) group. CSF metabolomics of medulloblastoma patients revealed significantly different profiles among LM diagnosed with different test results. We suggest that LM patients could be screened by appropriately selected LOME-generated LMIs to support LM diagnosis by either MRI or cytology alone.

## 1. Introduction

Medulloblastoma is the most common type of malignant central nervous system (CNS) tumors and accounts for 14% of causes of death in primary brain tumors in 0–14 year-old children [1,2]. Medulloblastoma occurs in the cerebellum and 4th ventricle and frequently disseminates into cerebrospinal fluid (CSF), and develops leptomeningeal metastasis (LM). Medulloblastoma LM in childhood patients remains a major cause of treatment failure. An initial treatment for medulloblastoma is provided according to the metastatic (M) stage determined by the results of both CSF cytology and magnetic resonance imaging (MRI), expecting an effective response and less complications in patients [3]. However, the false discovery in CSF cytology or MRI, a discord between those results, and the limited information on LM status are problems in the decision of medulloblastoma treatment [4,5,6,7,8].

The diagnostic accuracy improves when MRI and CSF cytology results concur with each other, but these results are frequently not in accordance [9,10]. The clinical meaning and biological relevance of discordant results have not been investigated. Both false negatives and false positives may have major adverse consequences, including life-threatening undertreatment or profound neurological/growth-related deficits, respectively.

Researchers have focused on CSF metabolomics and proteomics to identify biomarkers for diagnosis or disease activity [11,12]. Paine et al. recently evaluated differences in metabolomic profiles of tumor tissues according to metastasis status in medulloblastoma [13]. Advances in high-resolution mass spectrometry (MS) techniques have enabled the identification of small concentrations of metabolites in biofluids of cancer patients [14]. Previously, we elucidated discriminative metabolomic profiles according to varying CNS tumor statuses including LM from solid primary cancers in CSF of 196 patients [15].

Here, we reported both MRI findings and CSF cytology of each CSF sample from medulloblastoma patients and classified CSF samples into four groups: MRI (+)/cytology (+), MRI (+)/cytology (−), MRI (−)/cytology (+), and MRI (−)/cytology (−). Based on the assumption that treatments and LM-related symptoms could affect the LM diagnostic results, we further divided the samples into subgroups according to these factors. We assessed whether CSF metabolomic profiles could distinguish the different diagnostic result groups to improve our ability to diagnose LM in medulloblastoma. We also used low-mass ion (LMI) discriminant equation (LOME) algorithms to identify combinations of LMIs differentiating the groups and subgroups, suggesting that LOME may be a useful strategy to support the MRI and CSF cytology diagnosis of LM.

## 2. Results

### 2.1. Clinical Characteristics of Patients and CSF Samples

**Figure 1:**
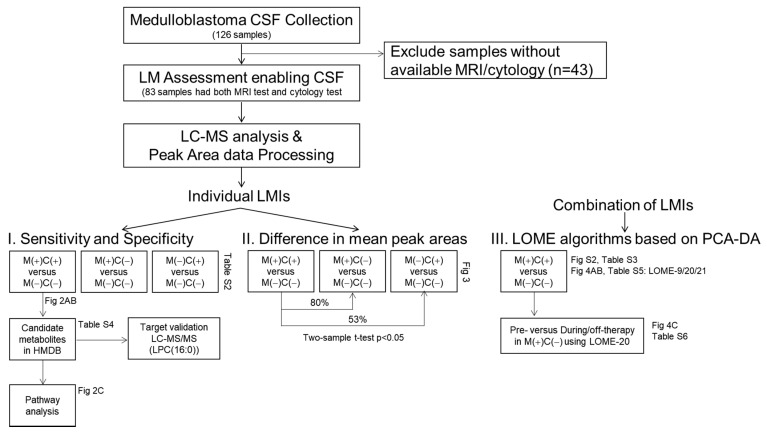
Study flow chart. Medulloblastoma CSF collection included 126 CSF samples from medulloblastoma patients, in which 83 samples possible to review with clear MRI and cytology test results were subjected to CSF metabolomic analyses. After peak area data processing, LMIs were profiled either single or in combination of sets.

Our study workflow was summarized in Figure 1. A total of 126 CSF samples were collected from medulloblastoma patients, and among them, 83 CSF samples from 45 patients were included to explore CSF metabolomics. Clinical characteristics of the patients are summarized in Table 1, 22 males and 23 females with age ranging from 2.8 to 24.4 years old. The CSF sampling conditions varied in this retrospective study (Supplementary Materials Table S1). Among 83 samples, 53 CSF samples were obtained via lumbar puncture, 25 samples were collected from a ventricle via an extraventricular drainage catheter, and 5 samples were obtained from the cisternal space either intraoperatively or postoperatively. The timing of CSF sampling in reference to adjuvant radiation or chemotherapy was as follows: pre-treatment, 37 samples; during treatment, 33 samples; and off-treatment (during follow-up), 13 samples.

### 2.2. CSF Cytology and MRI Findings for Diagnosis of LM

**Figure 2:**
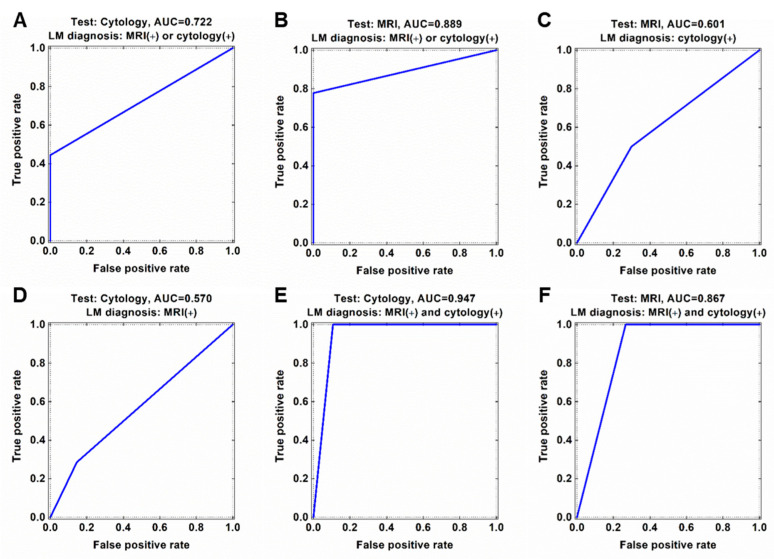
Discordance of CSF cytology and MRI findings for diagnosis of leptomeningeal metastasis (LM). Diagnostic accuracy in terms of area under the ROC curve (AUC) of (**A**) CSF cytology and (**B**) MRI based on current LM diagnosis criteria in medulloblastoma (either MRI or cytology positive). (**C**) ROC curve of MRI based cytology-preferred LM diagnosis. (**D**) ROC curve of cytology based on MRI-preferred LM diagnosis. ROC curve of (**E**) cytology and (**F**) MRI based on both MRI and cytology positive LM diagnosis.

Of the 83 analyzed CSF samples, eight samples were both CSF cytology (+) and MRI (+) and 47 samples were both cytology (−) and MRI (−) for LM (Table 2 and Supplementary materials Table S1). Tests for LM were MRI (+)/cytology (−) in 20 samples and MRI (−)/cytology (+) in the remaining 8 samples. When we assume all CSF samples with at least one positive finding either MRI or CSF cytology to represent true LM, both tests showed a low sensitivity (77.8% and 44.4%, respectively) and 100% specificity for diagnosing LM. The area under the receiver operating characteristics curve (AUC) was 0.722 for CSF cytology and 0.889 for MRI (Figure 2A,B). If cytology provided the true value for LM, the calculated diagnostic scores for MRI were sensitivity, 50%; specificity, 70.1%; and AUC, 0.601 (Figure 2C). Conversely, if MRI provided the true values for LM, the diagnostic scores for cytology were sensitivity, 28.6%; specificity, 85.5%; and AUC, 0.570 (Figure 2D). If we restricted the diagnosis of true LM to requiring positive results on both tests, then the specificity decreased. The AUC was 0.947 for both cytology and MRI (Figure 2E,F), but the specificity was 89.3% for CSF cytology and 73.3% for MRI.

### 2.3. Candidate Individual LMIs Discriminating between MRI (+)/Cytology (+) and MRI (−)/Cytology (−) Groups

**Figure 3:**
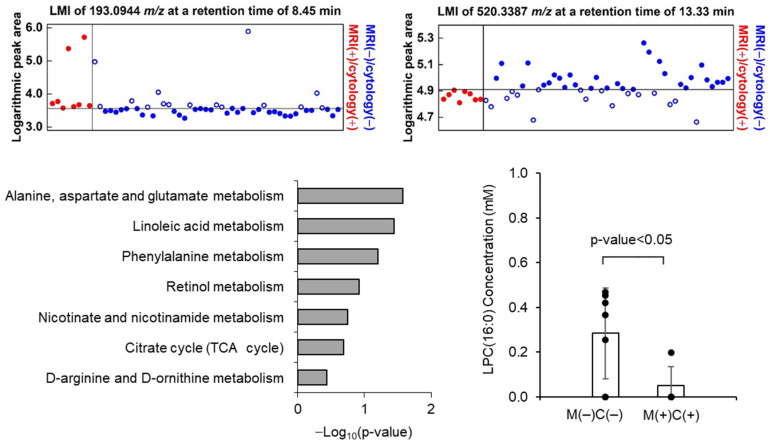
Example of discriminative low-mas ion (LMI) showing the exclusiveness at summed sensitivity and specificity of >160%, which (**A**) increased and (**B**) decreased LMI in MRI (+)/cytology (+) group compared to MRI (−)/cytology (−) group. (**C**) Candidate metabolites corresponding 27 LMIs were identified from Human Metabolite Database and the metabolic pathway enrichment analysis was performed from Metaboanaylst (ver. 4.0). *p*-value was calculated based on the global test statistics. (**D**) The expression level lysophosphatidylcholine (lysoPC (16:0)), which was validated through targeted MS/MS analysis, was significantly different between MRI (+)/cytology (+) and MRI (−)/cytology (−) CSF samples.

A total of 6572 LMIs were measured by LC-MS in the 83 CSF samples, each LMI was identified with their mass value (*m*/*z* ratio) and retention time (min), and the relative expression levels of LMIs were denoted by normalized peak areas. Based on the assumption that discriminative LMIs between MRI (+)/cytology (+) and MRI (−)/cytology (−) samples reflect LM activity, we firstly evaluated the logarithmic peak area of each LMI to assess whether it was expressed differently between the two groups. Among these LMIs, 27 LMIs were identified to discriminate MRI (+)/cytology (+) from MRI (−)/cytology (−) groups or vice versa with >160% of summed sensitivity and specificity (Supplementary materials Table S2, and Figure 2B and Figure 3A). Among these 27 discriminative LMIs, 23 LMIs (19 were highly expressed in MRI (+)/cytology (+) group, and so 4 were in MRI (−)/cytology (−) group) were identified to candidate molecules in the Human Metabolome Database (HMDB) (Table S3). We further determined the involved metabolomic pathways represented by these discriminative LMIs in MetaboAnalyst (www.metaboanlyst.ca, version 4.0, accessed on 19 November 2020). Pathways were those related to the metabolism of linoleic acid, nicotinate-nicotinamide, retinol, phenylalanine, arginine/ornithine, and alanine/aspartate/glutamate, as well as the tricarboxylic acid cycle (Figure 3C).

To validate the candidate molecules identified from HMDB, we tentatively chose lysophophatidyl choline (lysoPC, *m*/*z* 520.3387) and confirmed that the MS/MS patterns of the candidate molecules (SigmaAldrich, St. Louis, MO, USA, CAS no. 9008-30-4) are in accordance with the CSF LC-MS/MS patterns (Figure S2). Next, we verified the expression of lysoPC in MRI (+)/cytology (+) (*n* = 7) and MRI (−)/cytology (−) (*n* = 4) in available CSF as internal validation. As expected, lysoPC concentration of MRI (−)/cytology (−) (mean 0.285 mM, standard deviation ± 0.204) was significantly higher than that of MRI (+)/cytology (+) (mean 0.049 mM, ±0.204) (Figure 3D).

### 2.4. Comparisons of LMI Profiles According to MRI and Cytology Results

Based on the assumption that LMIs discriminating between MRI (+)/cytology (+) and MRI (−)/cytology (−) are byproducts of LM activity, we evaluated whether discriminative LMIs were also significantly differentially expressed in the MRI (+)/cytology (−) and MRI (−)/cytology (+) groups, compared with the MRI (−)/cytology (−) group. We identified that among 27 discriminative LMIs (>160% of summed sensitivity and specificity), 9 LMIs in the MRI (+)/cytology (−) group and 12 LMIs in the MRI (−)/cytology (+) group with (Table S2).

**Figure 4:**
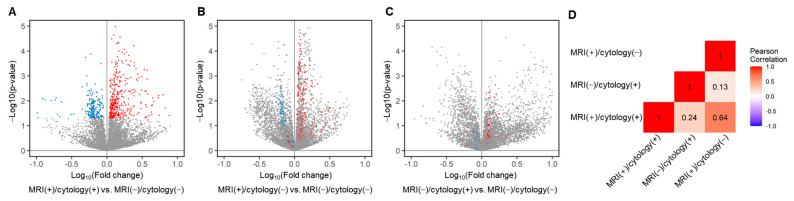
Volcano plot depicting the mean peak area of each LMIs compared between each LM groups and MRI (−)/cytology (−) groups. (**A**) The LMIs showed significantly different expression between MRI (+)/cytology (+) group and MRI (−)/cytology (−) group. The two-sample *t*-test *p*-value of <0.05 was considered significant (579 LMIs, blue, decreased; red, increased). (**B**) The LMIs showed significantly different expression between MRI (+)/cytology (−) group and MRI (−)/cytology (−) group. (**C**) The LMIs showed significantly different expression between MRI (−)/cytology (+) group and MRI (−)/cytology (−) group. X: Fold change in the normalized peak area of the indicated group compared to MRI (−)/cytology (−) group, Y: −log10 (*p*-value) between two groups. (**D**) The Pearson’s correlation analysis was calculated and the heatmap was drawn in R (ver. 4.0.4), using the mean peak area of 579 LMIs of (**A**).

Additionally, we evaluated the difference in mean peak area of each LMI between the three LM-positive groups and the MRI (−)/cytology (−) group. In MRI (+)/cytology (+) group, 195 LMIs significantly decreased and 384 LMIs significantly increased (Figure 4A). The MRI (+)/cytology (−) group had 512 of significantly decreased LMIs and 498 of significantly increased LMIs, whereas the MRI (−)/cytology (+) group exhibited with 377 of significantly decreased LMIs and 331 of significantly increased LMIs (Figure 2C and Figure 4B). The number of differentially abundant LMIs were different between three LM groups.

We also traced how the discriminative LMI profiles of the MRI (+)/cytology (+) group were expressed in the MRI (+)/cytology (−) and MRI (−)/cytology (+) groups (colored dots in Figure 4B,C). In the MRI (+)/cytology (−) group, 33.8% (66/195) of the decreased LMIs in the MRI (+)/cytology (+) group were still significantly decreased, and 30.7% (118/384) of the increased LMIs remained significantly increased. By contrast, in the MRI (−)/cytology (+) group, 1.0% (2/195) of the decreased LMIs in the MRI (+)/cytology (+) group were also significantly decreased, but only 4.2% (16/384) of the increased LMIs remained significantly increased. Thus, the proportion of remaining decreased/increased MRI (+)/cytology (+) LMIs differed significantly between the MRI (+)/cytology (−) and MRI (−)/cytology (+) groups (*p* = 0.0012). The correlation of these differently expressed LMIs showed a correlation between MRI (+)/cytology (+) and MRI (+)/cytology (−) groups (Pearson correlation, r = 0.64), whereas there was no correlation of differentially expressed LMIs between MRI (+)/cytology (+) and MRI (−)/cytology (+) groups (r = 0.24) (Figure 4D). These results suggest that the LMI profiles of the MRI (+)/cytology (−) group were more similar to those of the MRI (+)/cytology (+) group than those of the MRI (−)/cytology (+) group.

### 2.5. LOME Discriminating Different MRI and Cytology Results for LM, Sampling Times, and Symptoms Status

**Figure 5:**
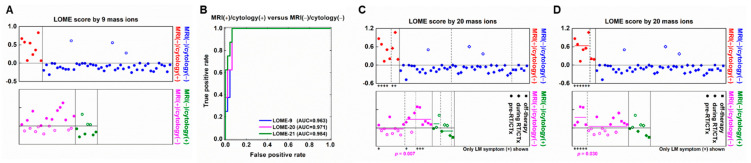
Selection of LM discriminant low-mass ions by low-mass ion discriminant equation (LOME). (**A**) LOME-9 distinguishing the MRI (+)/cytology (+) group and MRI (−)/cytology (−) group. (**B**) The area under the receiver operating characteristics curve of LOME-9. (**C**) LOME distinguishing significantly the sampling time (*p* = 0.007), and (**D**) the presence of LM-related symptoms (*p* = 0.030) in MRI (+)/cytology (−) group. The + sign below the box indicates LM symptom positive CSF samples. LOME-20 was also constructed to distinguish MRI (+)/cytology (+) from MRI (−)/cytology (−) groups. MRI (+)/cytology (−) and MRI (−)/cytology (+) samples were reclassified into MRI (+)/cytology (+) or MRI (−)/cytology (−) category by using the same LOME calculation process. A sample with a positive or negative LOME score was assigned as a predicted MRI (+)/cytology (+) or MRI (−)/cytology (−). Red solid symbols and blue hollow symbols respectively denote MRI (+)/cytology (+) and MRI (−)/cytology (−) samples predicted as MRI (+)/cytology (+), and blue solid symbols denote MRI (−)/cytology (−) samples predicted as MRI (−)/cytology (−). Magenta solid symbols and olive hollow symbols denote MRI (+)/cytology (−) and MRI (−)/cytology (+) samples predicted as MRI (+)/cytology (+), and magenta hollow symbols and olive solid symbols denote MRI (+)/cytology (−) and MRI (−)/cytology (+) samples predicted as MRI (−)/cytology (−).

PCA-DA on all 6572 LMIs yielded a sensitivity of 100% and specificity of 55.32% (Figure S3A). Search algorithm 1 identified 660 LMIs that contributed substantially to the PCA-DA score, and the results of this algorithm had a sensitivity of 100% and specificity of 59.57% (Figure S3B). In search algorithm 2, LOME selected 9 LMIs, the combination of which (LOME-9) had a specificity of 93.6% and sensitivity of 100% for differentiating MRI (+)/cytology (+) from MRI (−)/cytology (−) (Figure 2B and Figure 5A, and Table S4). These results suggest that LMI profiles may have a higher sensitivity and specificity for LM diagnosis than either MRI or cytology.

We also searched for combinations of discriminative LMIs that could differentiate between different MRI and cytology results, as well as between sampling time or between the presence or absence of LM-related symptoms (Table S5). Using search algorithm 2, we identified 20 LMIs, the combination of which (LOME-20) had a specificity of 93.6% for differentiating MRI (+)/cytology (+) from MRI (−)/cytology (−). LOME-20 also significantly differentiated the pre-treatment subgroup from the during/off-treatment subgroup (*p* = 0.007, Figure 5C) and the presence of LM-related symptoms from the absence of LM-related symptoms subgroup in the MRI (+)/cytology (−) group (*p* = 0.030, Figure 5D). Nevertheless, search algorithm 2 failed to identify a LOME that could distinguish between sampling time subgroups in the MRI (−)/cytology (+) group or between the presence or absence of LM-related symptoms subgroups in the MRI (+)/cytology (+) group at a *p* < 0.05 significance level, while maintaining the discriminant scores between MRI (+)/cytology (+) and MRI (−)/cytology (−) groups.

## 3. Discussion

### 3.1. Difficulties with Diagnosing LM in Medulloblastoma

As distant metastasis via CSF is one of the most important prognostic factors in medulloblastoma, treatment strategies depend on M staging, which is currently considered M1 or higher if either CSF cytology or MRI findings show evidence of LM. However, pathologists often report CSF cytology results as “suggestive of malignant cells” or “atypical cells present”. Radiologists often report MRI finding as “suspicious” or “equivocal” for LM. LM is frequently asymptomatic despite definite MRI findings of LM [10,16]. To avoid these diagnostic difficulties, both central review and detailed prospective protocols for CSF cytology and MRI acquisition are strongly recommended [17]. In the current retrospective study, the authors worked together while reviewing the MRI and CSF cytology results of all included cases, and each result was reported as positive or negative based on the consensus of the group.

Many studies have suggested that CSF cytology results can be affected by sampling conditions, including CSF volume, location, and timing in relation to surgery [6,7,8,10]. Response Assessment in Pediatric Neuro-Oncology (RAPNO) working group cautioned that intraoperative CSF sampling should be avoided, as it may lead to false positives from floating cells spilled during surgery [17,18]. However, preoperative lumbar puncture could be dangerous in children with a large posterior fossa mass. Thus, CSF cytology is frequently obtained intraoperatively or postoperatively.

The timing of spinal MRI is important, and M staging is ideally performed using a preoperative MRI. However, children with a posterior fossa tumor frequently present with emergent complications, such as hydrocephalus or brainstem compression, and sometimes require immediate surgery, before a spinal MRI can be obtained. Children’s Oncology group ACNS 9961 study suggested the importance of imaging techniques [19,20], as patients with inadequate staging images had a poorer prognosis than those with fully assessable images. Recently, RAPNO working group suggested standardized imaging protocols, timing of scans, and radiographic standards for evaluating LM in medulloblastoma [17].

### 3.2. Discordance between MRI Finding and CSF Cytology

The results of our study may help to optimize the diagnosis of LM for situations when MRI and CSF cytology findings are discordant. Meyers et al. reported the diagnostic accuracy of postoperative spinal MRI and CSF cytology with respect to medulloblastoma dissemination during follow-up, and MRI had a better sensitivity for the disease than cytology [7]. This result is in accordance with our study. Our CSF metabolomic profiles supported this finding, as discriminative LMIs for MRI (+)/cytology (+) more frequently remained significant in MRI (+)/cytology (−) cases than in MRI (−)/cytology (+) cases. In our previous study of non-small cell lung cancer patients with CSF cytology positive for LM, MRI findings of LM were suggestive of more advanced disease, as patients with these findings had poorer survival than those with an MRI negative for LM [10]. The prognostic differences in discordant diagnostic test results are already reflected in M staging. In many studies, patients with M1 disease, reflecting only positive CSF cytology, had better progression-free and overall survival than patients with M2 or M3 disease, indicative of dissemination on intracranial or spinal MRI images, respectively [9]. However, the significance of positive CSF cytology results in patients with M2 and M3 disease has not yet been evaluated with respect to LM in medulloblastoma. It is especially important to recognize that intraoperative and early postoperative CSF cytology results may represent false positives. Meyers et al. reported follow-up results of five medulloblastoma patients with positive CSF cytology but negative spinal MRI scans, and all three patients with positive cytology in the early postoperative period had no evidence of LM at a mean follow-up period of 3.5 years [7].

### 3.3. Importance of CSF Analysis and LOME Models

Both M stage and progression were based not on analysis of tumor tissue but on MRI and CSF findings. CSF is expected to express biomarkers more accurately than serum, as CSF directly contacts CNS tumors. Furthermore, although obtaining CSF requires a somewhat invasive procedure, serial samples can be collected, whereas it would be difficult or impossible to obtain tumor tissue samples at each suspicious area or timing of progression.

However, technical barriers exist between identifying discriminative LMIs and applying this technology as a diagnostic tool. One difficulty relates to the methods for determining discriminative LMIs. In this study, we used two different methods: one was based on exclusiveness, and the other was based on mean peak area differences between comparative groups. Until now, no standardized methods have been recommended for extracting discriminative LMIs in terms of such factors as MS conditions, absolute threshold levels, and statistical methods. Furthermore, we assume that the exclusiveness of discriminative LMIs cannot be maintained if more samples are added unless the LMI is a unique metabolite generated by LM progression. In this study, we identified LOME-9 and its sensitivity was superior to that of both MRI and cytology. LOME-20 revealed the possibility of not only differentiating patients with discordant CSF cytology and MRI results for LM from those with negative results on both tests, but also differentiating between sampling times or presence or absence of LM-related symptoms. These results suggest that selection of appropriate LMIs could contribute to the diagnosis of LM when MRI and CSF cytology results are discordant.

### 3.4. Limitations

The limitation of this study is that we were unable to evaluate bias from confounding factors, such as histologic or molecular subgroups, which may have affected the metabolomic profiles [21,22]. Furthermore, our identification of LMI metabolites using non-targeted metabolomics only depends on Human Metabolome Database information and was not supported by tandem MS patterns of standard metabolites. Our LMIs cannot be considered as specific molecules at this level unless verified by targeted MS. Thus, it will be essential to perform future experiments using targeted MS/MS based on our discriminative LMIs for LM, and prove the efficacy of LOME in external validation with a large number of CSF samples.

## 4. Materials and Methods

### 4.1. CSF Collection

All patients were signed informed consent and the study was approved by Institutional Review Board (Identifier: 2014-0135) of the National Cancer Center, Goyang, Republic of Korea, according to the ethical guidelines outlined in the Declaration of Helsinki. The informed consent was obtained from all patients and legal representatives for patients below 18 years. The CSF samples were collected from a ventricle either intraoperatively via an extraventricular drainage catheter or postoperatively via a shunt reservoir or from the thecal sac via lumbar puncture. To remove cells and debris, CSF sample was centrifuged at 2000× *g* for 20 min within 1 h of collection and the supernatant was further centrifuged at 10,000× *g* for 30 min. Finally, the supernatant was aliquoted and frozen at −80 °C for future investigations.

### 4.2. Inclusion Criteria

We collected 126 medulloblastoma CSF samples between 2015 and June 2019 (Table S1). After reviewing the electronic medical records and the results of MRI and CSF cytology tests obtained within 1 month of CSF sampling, we excluded 43 samples without clear corresponding MRI or CSF cytology results at the time of CSF sampling. Thus, this study included 83 CSF samples for which there were both MRI and CSF cytology results and for which the diagnosis of medulloblastoma was confirmed histologically.

### 4.3. Diagnosis of Leptomeningeal Metastasis

Working together, the authors (G.H.S., P.H.J., P.M.L. and L.S.H.) reviewed the MRI and CSF cytology results of all cases to identify the presence of LM in a binary category. CSF cytology results with “positive” or “suggestive” findings were considered positive for LM, and those with “atypical” or “negative” findings were considered negative for LM. MRI images were considered positive for LM if they exhibited definite findings of LM, such as clear leptomeningeal enhancement of the cerebral hemisphere gyri, cerebellar folia, subependymal wall, spinal cord, and cauda equina, or if they showed findings suggestive of LM, such as ventricular seeding [23].

### 4.4. Metabolite Extraction from CSF

CSF metabolites were extracted using a modified Bligh and Dyer method, as previously described [15]. Briefly, 50 µL CSF was added to 1 mL water, 2 mL methanol, and 0.9 mL dichloromethane, and mixed thoroughly by vortexing. The mixture was incubated on ice for 30 min, and added again to 1 mL water and 0.9 mL dichloromethane, followed by centrifuging at 1000× *g* for 10 min at room temperature. Nitrogen gas was used to dry the supernatant for LC-MS analysis.

### 4.5. Discovery of Individual Discriminative Low-Mass Ions

Samples were subjected to untargeted LC-MS measurements using a TripleTOF 5600+ system (Sciex, Framingham, MA, USA) and MarkerView software (version 1.2.1, Sciex) [24]. The peaks table consisted of one normalized peak area column per sample, as well as a mass value (*m*/*z*, mass-to-charge ratio) and retention time (min) column common to all samples. The nonzero peak areas were converted to common logarithms. LMIs with good discriminative ability (e.g., distinguishing MRI (+)/cytology (+) from MRI (−)/cytology (−) groups) were identified using the logarithmic peak area. The procedures for assessing individual LMIs were as follows. (1) For each LMI, a discrimination threshold value was determined (using increments of 0.01), wherein the sum of the sensitivity and specificity was highest (if adjacent threshold values had the same highest discrimination performance, the mean value was used). (2) Individual LMIs showing good discrimination (a summed sensitivity and specificity of 160% or higher) were set aside for subsequent analysis. The same procedures as above were applied to discover individual LMI candidates for distinguishing MRI (+)/cytology (−) or MRI (−)/cytology (+) from MRI (−)/cytology (−) groups, after replacing the MRI (+)/cytology (+) group with the MRI (+)/cytology (−) group or MRI (−)/cytology (+) group.

### 4.6. Group Comparison of Individual Discriminative Low-Mass Ions

For the comparison of individual LMIs in different diagnostic test LM groups, the peak areas were converted to common logarithms and calculated the average of individual LMIs. To test the statistical significance, the two-sample t-test were employed following f-test testing the distribution normality. We also applied the false discovery rate-adjusted ***p***-value, a technique reducing error when conducting multiple comparison in many omics data analyses, however, we dismissed it because few LMIs remained. For the correlation analysis, the average of individual LMIs were compared between different LM groups. All computational analyses were performed using R software (version 3.6.2 or 4.0.4, Boston, MA, USA).

### 4.7. Target Validation by LM-MS/MS

Using metabolite databases comprising Human Metabolome Database (HMDB), specific compounds were found for the given *m*/*z*, listed in rank order based on the MS and MS/MS data. The standard material of the selected specific compound was purchased from Sigma-Aldrich and targeted metabolomic analysis was performed by LC-MS/MS system to confirm whether the MS and MS/MS spectra of the standard material match the information of the selected specific compound. Targeted metabolomic analysis were performed using Shimadzu LC 40 system equipped with binary pump, degasser, column oven, and autosampler and coupled by means of an electrospray ion source (Turbo Spray ion source, AB Sciex) to an AB Sciex Triple Quad 5500+ mass spectrometer. Quantitative comparative analysis of specific metabolites in the CSF of cancer patients and non-patients was confirmed through targeted metabolic analysis with the standard curve of the standard material.

### 4.8. Low-Mass ion Discriminant Equation (LOME) Discriminating MRI (+)/Cytology (+) from MRI (−)/Cytology (−)

A discriminative combination of LMIs was searched by constructing a LOME consisting of two search algorithms based on principal component analysis–based discriminant analysis (PCA-DA), as previously described [15]. The detailed method is available in supporting file (Supplementary Materials and Figure S1).

### 4.9. Statistical Analyses

The independent two-sample t test was performed to compare mean scores between the pre-adjuvant treatment and during/off-treatment subgroups within the MRI (+)/cytology (−) and MRI (−)/cytology (+) groups and between the presence or absence of LM-related symptoms subgroups in the MRI (+)/cytology (+) and MRI (+)/cytology (−) groups. ***p***-values < 0.05 were considered indicative of a statistically significant result. All statistical analyses were performed using R software (version 3.6.2 or 4.0.4, Boston, MA, USA).

## 5. Conclusions

CSF metabolomic profiles from non-targeted MS analysis in medulloblastomas revealed significantly different profiles according to different MRI and CSF cytology results for LM. Our findings suggest that LMI profiles may have higher sensitivity for LM diagnosis than either MRI or cytology alone, when a combination of appropriate discriminant LMIs are selected using LOME. A future validation trial with targeted MS could be based on the candidate LMIs identified in this study.

## Figures and Tables

**Table 1:** Clinical characteristics of patients with medulloblastoma (*n* = 45).

	Number
Median age (range)	9.9 (2.8–24.4)
Gender	
Male	22
Female	23
Primary	
Primary	41
Recurrent	4
Histologic subtype	
Classic	31
Large cell/anaplastic	7
Desmoplastic/nodular	5
Extensive nodularity	2
M stage (*n* = 41)	
M0	16
M1	7
M2	6
M3	12
Residual tumor (*n* = 41)	
≥1.5 cm^2^	14
<1.5 cm^2^	3
No gross residual	24
Risk group (*n* = 41)	
Average risk	13
High risk	28

**Table 2:** Correlation of MRI leptomeningeal metastasis finding and CSF cytology.

CSF Cytology	MRI		Total
	(+)	(−)	
(+)	8	8	16
(−)	20	47	67
Total	16	55	83

Abbreviations: CSF, cerebrospinal fluid; MRI, magnetic resonance image.

## Data Availability

The datasets generated during and/or analyzed during the current study are available from the corresponding author on reasonable request. The data are not publicly available due to the information that could compromise research participant consent.

## Supplementary Materials

The following are available online at https://www.mdpi.com/article/10.3390/metabo11120851/s1, Figure S1: Search algorithm 2, Figure S2: Targeted MS/MS of candidate molecule, lysophosphatidylcholine (lysoPC (16:0)), Figure S3: Principal component analysis-based discriminant analysis (PCA-DA) with (A) total 6572 low-mass ions (LMIs) to distinguish MRI (+)/cytology (+) from MRI (−)/cytology (−) groups and (B) 660 LMIs chosen by search algorithm 1, Table S1: Different CSF sampling conditions of individual samples, Table S2: LMIs revealed different expression level between groups of different MRI and cytology result at a summed sensitivity and specificity > 160%, Table S3: Candidate molecules representing both MRI (+)/cytology (+) group in reference to MRI (−)/cytology (−) groups known to have similar *m*/*z* with LMI. Table S4: List of low-mass-ions (LMIs) selected by algorithm of the highest sensitivity with the lowest LMI number, Table S5: Differences in averages between the sampling time of pre-adjuvant treatment and during/off-treatment or between the presence and absence of LM-related symptoms.
