# Supplementary material for: Experimental Assessment of Leptomeningeal Metastasis Diagnosis in Medulloblastoma Using Cerebrospinal Fluid Metabolomic Profiles
Source: Metabolites. 2021 Dec 7;11(12):851. doi: 10.3390/metabo11120851 (PMC8708677; doi:10.3390/metabo11120851)
Supplement: Supplementary file 1 [file metabolites-11-00851-s001.zip › Metabolites_Supplementary Materials (R1).pdf]

Supplementary Materials

Supplementary Materials and Methods

Low-mass ion discriminant equation discriminating MRI(+)/cytology(+) from MRI(-)/cytology(-)

We searched for a discriminative combination of LMIs by constructing a LOME consisting of two search algorithms based on principal component analysis–based discriminant analysis (PCA-DA). *Search algorithm 1:* PCA-DA, the same analysis module as the MarkerView software, requires an aligned mass spectra (a peak area table). The nonzero peak areas in the normalized peaks table were converted to common logarithms, after which Pareto-scaling was performed. The PCA-DA score was the weighted sum of the Pareto-scaled peak areas of all detected LMIs. However, only a small portion of the LMIs (i.e., numerically significant LMIs) dominated the PCA-DA score. Search algorithm 1 was applied to the PCA-DA results to reveal a candidate set of *P* LMIs based on two requirements: weighted peak area >0.01 for each sample and identified in more than half of all samples. A peaks table of only numerically significant *P* LMIs was constructed and the PCA-DA of the peak areas in the reduced peaks table was performed again to update weighting factors for the numerically significant *P* LMIs. *Search algorithm 2:* A discriminative set of LMIs was identified among *P* LMI candidates with search algorithm 2, consisting of germination (Supplementary Figure 1A and 1D), growth (Supplementary Figure 1B and 1D), and shrinkage (Supplementary Figure 1C and 1D) modules. A seed set was obtained from the germination module, after which the growth and shrinkage modules were alternately performed on the seed set until there was no further improvement in discriminant performance (Supplementary Figure 1E). The final upgraded set was designated as the discriminative set of LMIs. There was also a combination strategy that did not include the shrinkage module and iterative process. The iterative process was initiated by applying the growth or shrinkage module to the seed set. These three combination strategies of modules and

some seed sets produced several discriminative sets of LMIs.

The germination module was constructed using six steps: 1) we first inspected the individual LMIs to determine whether any had a sensitivity and specificity of 100%; 2) the sums of the sensitivity and specificity for ${}_{P}C_2$ and ${}_{P}C_3$ LMI combinations were calculated; 3) the two- or three-LMI set with the highest summed sensitivity and specificity was set aside and step 2 was repeated with the remaining LMIs until only one LMI remained; 4) any two- or three-LMI set was considered as a single LMI and steps 2) to 3) were repeated; 5) step 4 was repeated, with the LMI set created at the previous stage considered as a single LMI at the next stage; and 6) the set of S LMIs with the highest summed sensitivity and specificity was designated as the seed set. This last step was applied to stage 2 or higher (Supplementary Figure 1A and 1D), leading to multiple seed sets.

The growth module was created using these additional steps: 7) the seed set was augmented with the ${}_{R}C_1$, ${}_{R}C_2$, and ${}_{R}C_3$ LMI combinations, in which $R=P–S$; 8) the augmented seed set with the highest summed sensitivity and specificity was designated as a new seed set if its summed sensitivity and specificity was greater than that of the current seed set, and step 7 was repeated with the remaining LMIs; and 9) the last updated seed set was considered the discriminative set of LMIs.

The shrinkage module was constructed in a similar, but opposite, manner to that used for the growth module. It consisted of these steps: 10) the seed set was reduced by the ${}_{S}C_1$, ${}_{S}C_2$, and ${}_{S}C_3$ LMI combinations; 11) the reduced seed set with the highest summed sensitivity and specificity was designated as a new seed set if its summed sensitivity and specificity was greater than that of the current seed set, and step 10 was repeated; and 12) the last updated seed set was designated as the discriminative set of LMIs.

In the above modules, when more than one LMI set had the same highest summed sensitivity and specificity, one LMI set was selected in this manner: when the number of LMIs in the sets

with identical summed sensitivity and specificity values was not the same, the set with the fewest LMIs was selected, whereas when the number of LMIs in the sets was identical, the set with the highest Fisher's discriminant ratio was selected.

LOME construction and evaluation: In summary, search algorithm 1 identified numerically significant *P* LMIs that contributed substantially to the PCA-DA score for distinguishing the MRI(+)/cytology(+) group from the MRI(−)/cytology(−) group. Then, search algorithm 2 revealed several LMI combinations among the numerically significant *P* LMIs by maximizing the summed sensitivity and specificity. Next, the MRI(+)/cytology(−) and MRI(−)/cytology(+) samples were reclassified into MRI(+)/cytology(+) or MRI(−)/cytology(−) categories using the same LMI combinations. In addition, the independent two-sample two-sided *t* test was used to compare mean LOME scores (weighted sum of Pareto-scaled peak areas of the selected LMIs) between the pre-adjuvant treatment and during/off-treatment subgroups within the MRI(+)/cytology(−) and MRI(−)/cytology(+) groups and between the presence and absence of LM-related symptoms subgroups in the MRI(+)/cytology(+) and MRI(+)/cytology(−) groups.

Table S1. Different CSF sampling conditions of individual samples.

Table S2. LMIs revealed different expression level between groups of different MRI and cytology result at a summed sensitivity and specificity > 160%.

Table S3. Candidate molecules representing both MRI (+)/cytology (+) group in reference to MRI (−)/cytology (−) groups known to have similar m/z with LMI.

Selected LMI (m/z)	HMDB identifier	Candidate metabolite	Chemical Formula
**68.9835**	HMDB0001525	Imidazole	C3H4N2
**102.1287**	HMDB0001252	Betaine aldehyde	C5H12NO
**116.0695**	HMDB0000162	L-Proline	C5H9NO2
	HMDB0012880	Acetamidopropanal (spermine derivative)	C5H9NO2
**118.0859**	HMDB0000883	L-Valine	C5H11NO2
	HMDB0000128	Guanidoacetic acid (glycine metabolite)	C3H7N3O2
**121.0838**	HMDB0001366	Purine	C5H4N4
**123.0836**	HMDB0001406	Nicotinamide (Vit. B3)	C6H6N2O
**124.0873**	HMDB0001488	Nicotinic acid	C6H5NO2
**133.1046**	HMDB0000214	Ornithine	C3H4O2
	HMDB0001624	2-Hydroxyisocaproic acid (leucine derivative)	C6H12O3
**137.0699**	HMDB0000209	Phenylacetic acid (phenylalanine derivative)	C8H8O2
	HMDB0000157	Hypoxanthine (purine derivative)	C5H4N4O
**157.016**	HMDB0000226	Orotic acid (Vit. B12)	C5H4N2O4
**193.0937**	HMDB0029048	Serylserine (serine dipeptide)	C6H12N2O5
**193.0944**	HMDB0028768	Cysteinyl-Alanine	C6H12N2O3S
	HMDB0000094	Citric acid	C6H8O7
**283.1759**	HMDB0029073	Threoninyl-Tyrosine	C13H18N2O5
**285.2108**	HMDB0001358	Retinal (Vit. A)	C20H28O
	HMDB0000827	Stearic acid	C18H36O2
**295.1905**	HMDB0028941	Leucyl-Tyrosine	C15H22N2O4
	HMDB0011108	17-Hydroxylinolenic acid	C18H30O3
**297.2416**	HMDB0004667	13-Hydroxyoctadecadienoic acid	C18H32O3
**305.1562**	HMDB0000309	3a,16b-Dihydroxyandrostenone	C19H28O3
	HMDB0060102	Arachidonic acid	C20H32O2
**333.2767**	HMDB0060407	5alpha-Dihydrodeoxycorticosterone	C21H32O3
	HMDB0000363	17a-Hydroxypregnenolone	C21H32O3
**363.1999**	HMDB0014879	Cortisol	C21H30O5
	HMDB0000319	18-Hydroxycorticosterone	C21H30O5
**372.2335**	HMDB0013658	Docosahexaenoyl ethanolamide	C24H37NO2
	HMDB0005066	Tetradecanoylcarnitine	C21H41NO4
**378.9007**	no result		
**429.3197**	HMDB0093320	Diacylglycerol (14:0/0:0/8:0)	C25H48O5
**446.8884**	no result		
**484.7768**	no result		
**486.1108**	no result		
**520.3387**	HMDB0010386	Lysophophatidylcholine	C26H50NO7P
**528.3086**	HMDB0011524	Lysophosphatidylethanolamine	C27H46NO7P

Note: LMIs of shaded background are increased in MRI (−)/cytology (−) group

Table S4. List of low-mass-ions (LMIs) selected by algorithm of the highest sensitivity with the lowest LMI number.

**LOME-9**		
**Mass value (m/z)**	**Retention time (min)**	**Discrimination**
118.0859	2.14	Specificity: 93.6% Sensitivity: 100.0%
126.0913	8.05	
132.1009	2.31	
147.0648	2.19	
174.1867	8.54	
202.0627	2.15	
258.2817	10.40	
498.9010	21.27	
506.3455	8.20	
**LOME-20**		
**Mass value (m/z)**	**Retention time (min)**	
82.0544	20.49	Specificity: 93.6% Sensitivity: 100.0%
118.0858	2.10	
118.0859	2.14	
118.1219	8.48	
120.0803	8.30	
126.0913	8.05	
132.1009	2.31	
147.0648	2.19	
202.0627	2.15	
219.2143	8.34	
372.2288	8.32	
498.8985	20.41	
498.9010	21.27	
506.3455	8.20	
523.3432	8.32	
570.3121	8.35	
586.3063	8.46	
654.4195	8.68	
779.8656	8.24	
853.7280	21.61	

Table S5. Differences in averages between the sampling time of pre-adjuvant treatment and during/off-treatment or between the presence and absence of LM-related symptoms.

1) Sampling time

	MRI(+)/cytology(−)			MRI(−)/cytology(+)		
	Average score			Average score		
	pre-adjuvant treatment	during/off-treatment	*t*-test *p*-value	pre-adjuvant treatment	during/off-treatment	*t*-test *p*-value
LOME-9	0.0230	0.1879	0.128	-0.0176	-0.1088	0.580
LOME-20	-0.0301	0.2819	0.006	0.1357	-0.0994	0.167

2) Presence of LM-related symptoms

	MRI(+)/cytology(−)			MRI(−)/cytology(+)		
	Average score			Average score		
	LM Symptom positive	LM Symptom negative	*t*-test *p*-value	LM Symptom positive	LM Symptom negative	*t*-test *p*-value
LOME-9	0.5079	0.1505	0.125	0.2686	0.0511	0.079
LOME-20	0.6313	0.1968	0.122	0.3499	0.0512	0.029

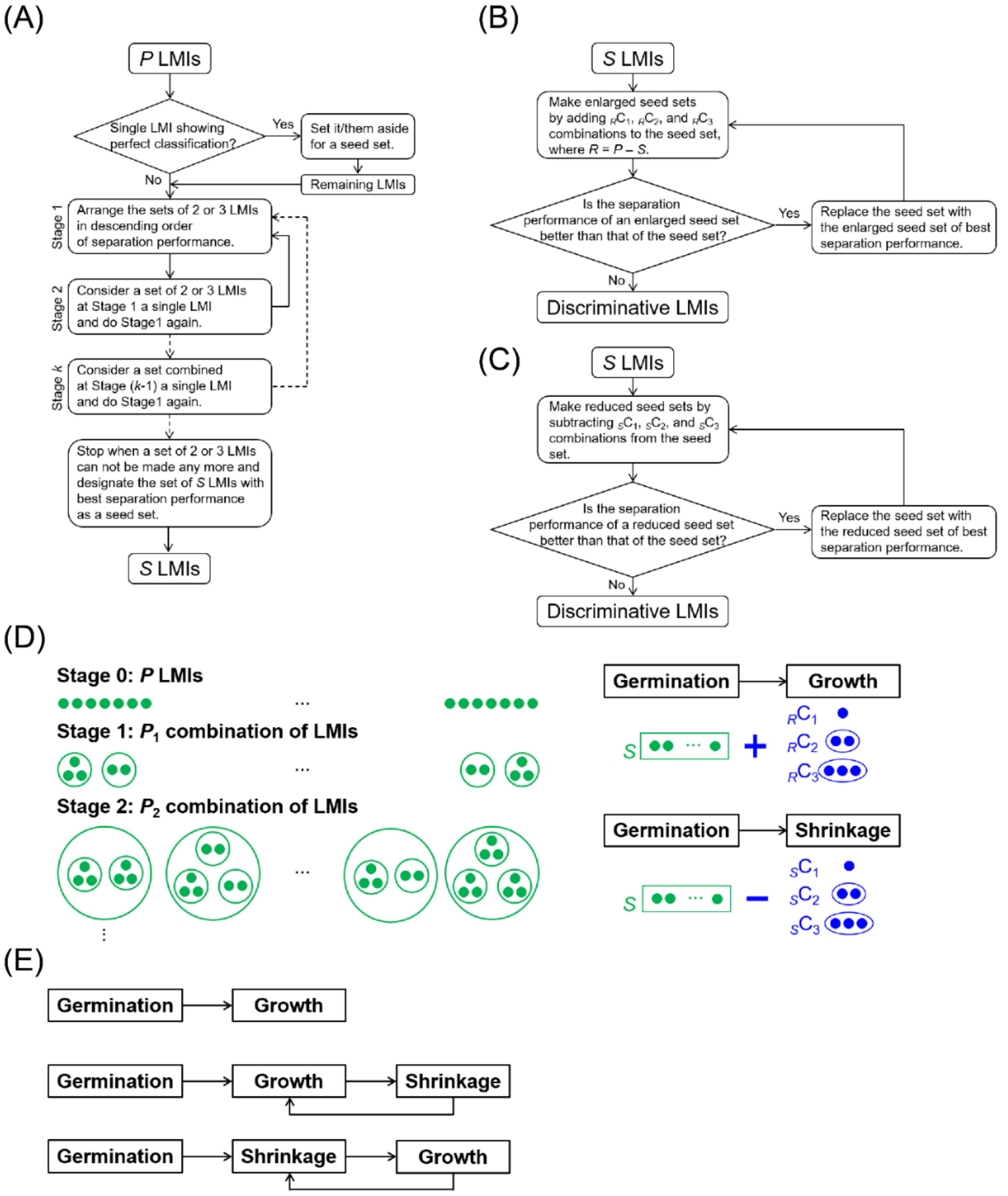

Figure S1. Search algorithm 2. (A) Germination module. (B) Growth module. (C) Shrinkage module. (D) Schematic drawings of the three modules. (E) Three combination strategies of the modules. Abbreviation; LMI, low-mass ion.

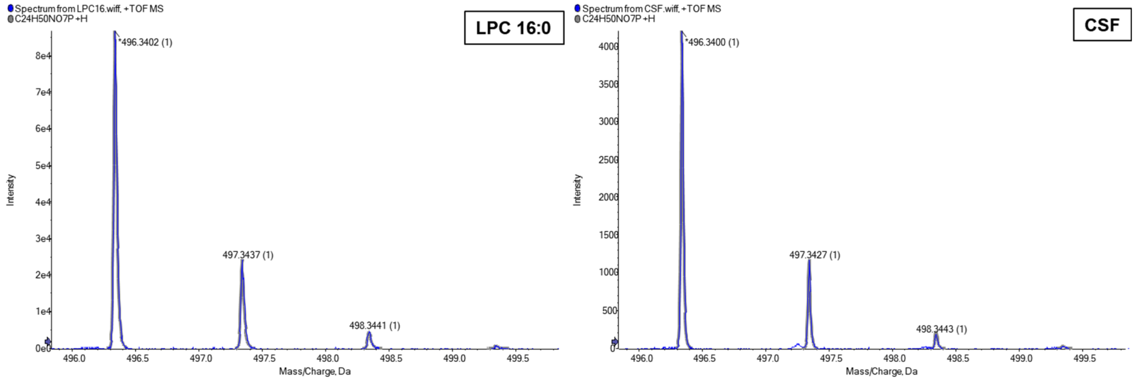

Figure S2. Targeted MS/MS of candidate molecule, lysophosphatidylcholine (lysoPC(16:0)). MS/MS peak of m/z 496.3400 (*left*) is observed at CSF MS/MS (*right*).

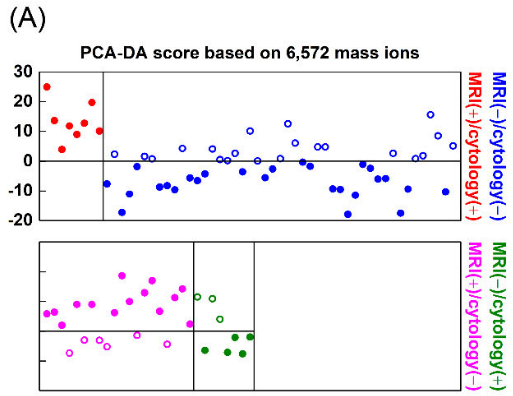

Figure S3. Principal component analysis-based discriminant analysis (PCA-DA) with (A) total 6,572 low-mass ions (LMIs) to distinguish MRI (+)/cytology (+) from MRI (−)/cytology (−) groups and (B) 660 LMIs chosen by search algorithm 1. MRI (+)/cytology (−) and MRI (−)/cytology (+) samples were reclassified into MRI (+)/cytology (+) or MRI (−)/cytology (−) category by using the same PCA-DA calculation process. A sample with a positive or negative PCA-DA score was assigned as a predicted MRI (+)/cytology (+) or MRI (−)/cytology (−). Red solid circles and blue hollow circles respectively denote MRI (+)/ cytology (+) and MRI (−)/cytology (−) samples predicted as MRI (+)/cytology (+), and blue solid circles denote MRI (−)/cytology (−) samples predicted as MRI (−)/ cytology (−). Magenta solid circles and olive hollow circles denote MRI (+)/cytology (−) and MRI (−)/cytology (+) samples predicted as MRI(+)/ cytology (+), and magenta hollow circles and olive solid circles denote MRI (+)/cytology (−) and MRI (−)/ cytology (+) samples predicted as MRI (−)/cytology (−).
